# Preparation of Progressive Antibacterial LDPE Surface via Active Biomolecule Deposition Approach

**DOI:** 10.3390/polym11101704

**Published:** 2019-10-17

**Authors:** Salma Habib, Marian Lehocky, Daniela Vesela, Petr Humpolíček, Igor Krupa, Anton Popelka

**Affiliations:** 1 Center for Advanced Materials, Qatar University, P.O. Box 2713, Doha, Qatar; salma.m.habib@hotmail.com (S.H.); igor.krupa@qu.edu.qa (I.K.); 2 Centre of Polymer Systems, Tomas Bata University in Zlin, Trida Tomase Bati 5678, 760 01 Zlin, Czech Republic; lehocky@post.cz (M.L.); dvesela@utb.cz (D.V.); humpolicek@utb.cz (P.H.); 3 Faculty of Technology, Tomas Bata University in Zlin, Vavreckova 275, 760 01 Zlin, Czech Republic

**Keywords:** biointerface, polyethylene, plasma treatment, antibacterial, grafting modification

## Abstract

The use of polymers in all aspects of daily life is increasing considerably, so there is high demand for polymers with specific properties. Polymers with antibacterial properties are highly needed in the food and medical industries. Low-density polyethylene (LDPE) is widely used in various industries, especially in food packaging, because it has suitable mechanical and safety properties. Nevertheless, the hydrophobicity of its surface makes it vulnerable to microbial attack and culturing. To enhance antimicrobial activity, a progressive surface modification of LDPE using the antimicrobial agent grafting process was applied. LDPE was first exposed to nonthermal radio-frequency (RF) plasma treatment to activate its surface. This led to the creation of reactive species on the LDPE surface, resulting in the ability to graft antibacterial agents, such as ascorbic acid (ASA), commonly known as vitamin C. ASA is a well-known antioxidant that is used as a food preservative, is essential to biological systems, and is found to be reactive against a number of microorganisms and bacteria. The antimicrobial effect of grafted LDPE with ASA was tested against two strong kinds of bacteria, namely, *Staphylococcus aureus* (*S. aureus*) and *Escherichia coli* (*E. coli*), with positive results. Surface analyses were performed thoroughly using contact angle measurements and peel tests to measure the wettability or surface free energy and adhesion properties after each modification step. Scanning electron microscopy (SEM) and atomic force microscopy (AFM) were used to analyze the surface morphology or topography changes of LDPE caused by plasma treatment and ASA grafting. Surface chemistry was studied by measuring the functional groups and elements introduced to the surface after plasma treatment and ASA grafting, using Fourier transform infrared (FTIR) spectroscopy and X-ray photoelectron spectroscopy (XPS). These results showed wettability, adhesion, and roughness changes in the LDPE surface after plasma treatment, as well as after ASA grafting. This is a positive indicator of the ability of ASA to be grafted onto polymeric materials using plasma pretreatment, resulting in enhanced antibacterial activity.

## 1. Introduction

Low-density polyethylene (LDPE) is the most common industrial polymer and is mainly used in food packaging because of its useful properties, such as its ease of shaping, handling, and recycling, and its high cost efficiency [1,2,3,4]. Nevertheless, LDPE lacks proficiency in traits such as printability, adhesion, and some other surface properties, as it has an inert surface with a very low surface free energy (wettability). Changing the bulk properties of LDPE by mixing additives is not recommended for food packaging applications; therefore, surface modification is a safe, easy, and cost-effective option [5,6]. Many studies have enhanced the surface properties of LDPE using physical or chemical methods together with conventional methods such as flame treatment, chemical grafting, irradiation, free-radical mechanism, and corona and radio-frequency (RF) plasma treatments [7,8,9,10,11]. These plasma treatments have been found to be more effective techniques for the modification of the surface properties of polymers without any disruption of bulk polymer properties [12,13]. Plasma, as the fourth state of matter, is able to initiate the ionization of air species and surface components, converting them into electrons and negative, positive, and neutral ions, in addition to metastable and free radicals [13,14,15]. The free radicals that are generated are readily reactive, with mainly oxygen-containing functional groups [16]. This results in enhancement of the wettability, adhesion, roughness, and reactivity properties. The plasma treatment methods vary from corona (atmospheric pressure) to vacuum-based RF plasma. All types were found to have similar effectiveness on the enhancement of the surface properties of polymers [17], even when the ionized gas species varied [18]. For nonthermal, low-temperature, and cold plasma, no heat is generated or required, and they only affect a layer of a few tens nanometers depth on the surface. Therefore, these methods are widely used in medical applications and medicine in general [16,19,20,21]. Plasma is also known to be safe for the environment and for human health, as it kills microorganisms and cleans medical equipment, with no significant impact on human cells [21,22,23].

In food packaging, microbial and bacterial fouling is a critical concern, and a large amount of research has been devoted to the use of antibacterial packaging materials to prevent the vulnerability and susceptibility of food to any type of microorganism and to increase the shelf life of the food [1,24,25]. Some antibacterial techniques involve mixing of the antibacterial agent within the polymeric material to generate biomaterials [26,27]. However, this process is not suitable for all packaging materials because it changes the main functional and mechanical properties of the materials and decreases their stability. Other studies have applied the antibacterial agents by surface grafting or surface tangling, with attachment on the surface achieved by chemical grafting, coating, or plasma treatment [28,29]. To enhance the antibacterial properties, different chemicals or nature-based compounds, such as chitosan [30,31,32,33] and alkyl pyridiniums [34], were grafted onto polymer surfaces and proved to reduce the number of, or entirely kill, bacteria (both gram-positive and gram-negative species). Other chemicals known as organic acids, which are mostly used as preservatives, were tested for their antibacterial activation when attached to polymers. Polyacrylic acid, for instance, was grafted with chitosan on LDPE and tested against the strong bacteria *Escherichia coli* (*E. coli*) [15]. These acids showed successful results in decreasing and eliminating the presence of bacteria on the LDPE surface.

Ascorbic acid (ASA), commonly known as vitamin C, is an organic acid known to be an antioxidant. It possesses two hydroxyl groups that can be deprotonated and is an effective radical scavenger [35,36]. It is an essential vitamin needed to maintain body health [37]. There are different studies on the antimicrobial effect of ascorbic acid that provide positive results. It was found to inhibit bacterial growth and prevent biological infections [38,39,40]. Further investigations found antiviral [40,41] as well as antifungal activities [40], alone or in combination with additional agents, to exert a synergetic effect. Tests with *E. coli*, *Staphylococcus aureus* (*S. aureus*), and some other types of bacteria [42,43,44,45] demonstrated the very effective ability of vitamin C to inhibit the growth of, and kill, bacterial colonies by penetrating bacterial walls, affecting the metabolism with no harmful effect on human cells [43]. ASA has the ability to enter a cell and modify its redox reaction through its hydroxyl groups, which eventually leads to the inhibition of microorganism growth; thus, ASA can be considered a good antimicrobial agent [46,47].

In this study, ASA was used for the preparation of a progressive LDPE surface through plasma-assisted grafting, which has excellent antimicrobial properties. The antibacterial effectiveness of the LDPE surface modified by ASA was tested against *E. coli* and *S. aureus*.

## 2. Materials and Methods

### 2.1. Materials

Commercial grade low-density polyethylene (LDPE) FE8000 was supplied in pellet form by Qatar Petrochemical Company (QAPCO, Doha, Qatar). Thin homogeneous films approximately 0.4 mm thick were prepared by compression molding using an industrial mounting press machine (Carver, Wabash, IN, USA). The pellets were melted at 160 °C and compressed for 2 min using a force of 2 tons, while maintaining the set temperature to obtain a film with the desired smooth surface. The samples were then cooled to room temperature by water. The LDPE films were cleaned by acetone to remove any additives, residuals, or any possible contaminations from the molding process that might affect the surface properties, and were then dried in an air atmosphere for 20 min at room temperature. Small strips (5 cm × 1 cm) were cut out and directly used for the surface treatment and subsequent analyses.

Ethylene glycol (> 98% FLUKA, Morris Plains, NJ, USA), formamide (> 98% FLUKA, Merelbeke, Belgium), ultra-pure water (prepared by Purification System Direct Q3, Millipore Corporation, Molsheim, France), and acetone (99.9% Scharlau, Barcelona, Spain) were used as testing liquids for wettability analyses.

L-ascorbic acid (> 99.0% Research-Lab, Uran Islampur, India) molecular weight = 176.14 g/mol was used as an antimicrobial agent.

### 2.2. Plasma Treatment of LDPE

Plasma treatment of LDPE films was performed using a Venus 75-HF enclosed low-temperature plasma-generating system (Plasma Etch Inc., Carson, CA, USA). Plasma-excited species were generated using a radio-frequency (RF) generator operating at a frequency of 13.56 MHz. The chamber of the plasma system was evacuated to a pressure level of approximately 0.2 Torr using a rotary vacuum pump before plasma ignition. Optimization of the treatment process was carried out by varying the nominal power, treatment time, and working gas to obtain the maximum level of hydrophilicity on the LDPE surface. The applied nominal power varied from 50 W to 120 W, and the treatment time ranged from 10 s to 180 s at a constant optimal nominal power of 80 W. The gas flow rate was 10 cm^3^/min. The film surfaces were treated from both sides in air.

### 2.3. Antibacterial Agent Grafting

**Figure 1:**
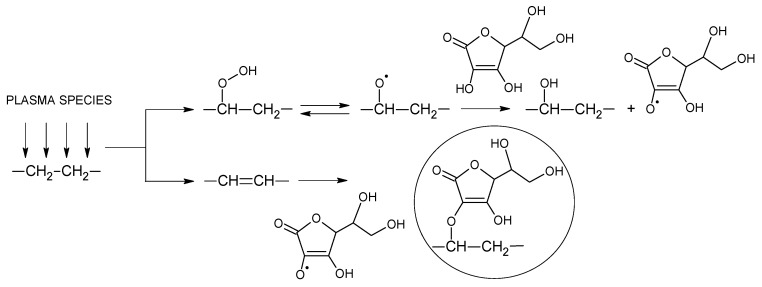
Scheme of ascorbic acid (ASA) grafting on low-density polyethylene (LDPE) via plasma treatment.

Immediately after the plasma treatment, the LDPE samples were immersed in a 10 vol % aqueous solution of ASA. The immersion process was continuous for 24 hours at 24 °C to achieve radical grafting. ASA is converted to an ascorbate radical by electron donation to a radical [48], namely, the alkoxyl radical present in the plasma-treated LDPE surface created by the decomposition of hydroperoxide. The ascorbate radicals can then interact with the double bonds present in plasma-treated LDPE created by disproportionation reactions; therefore, ASA can be covalently grafted onto the LDPE surface (Figure 1). After the grafting process, the LDPE samples were thoroughly washed with water and ethanol to remove weakly bound or unreacted ASA from the LDPE surface.

### 2.4. Hydroperoxide Determination

Iodometric titration was performed to determine the concentration of all hydroperoxide species accumulated on the surface of LDPE after plasma treatment. Plasma-treated LDPE samples were placed into a covered Erlenmeyer flask, which was filled with 50 ml of glacial acetic acid. An excess (1.0 g) of sodium iodide was added, and the flask was purged with argon gas for 15 min to eliminate interactions with air. After 15 min, the well-stirred mixture became yellow (oxidation of iodide to iodine by hydroperoxides incorporated on the LDPE surface) and was titrated with a 0.0005 M sodium thiosulfate pentahydrate aqueous solution. The reactions were carried out in an argon atmosphere and protected from light. The hydroperoxide concentration on the LDPE surface was calculated per area considering two treated sides of the LDPE samples. The titration was repeated 3 times to obtain average values and to ensure reliable results.

### 2.5. Surface Wettability Measurements

The changes in hydrophilicity induced by plasma treatment of LDPE films were evaluated by static contact angle measurements using the sessile drop method. An OCA35 surface free energy analysis system (DataPhysics, Filderstadt, Germany) equipped with a CCD camera was employed for this purpose. Water, formamide, and ethylene glycol were used as testing liquids to evaluate the total surface free energy and polar and dispersive components using the conventional Owens–Wendt–Rabel–Kaelble method. A droplet of approximately 3 µl of each testing liquid was placed on the air-facing samples. The contact angle was calculated after approximately 3 s to allow thermodynamic equilibrium between the liquid and the sample interface to be reached. The reported value for each testing liquid corresponds to the mean of at least five measurements taken on different parts of the substrate surface.

### 2.6. Graft Yield Analysis

Graft yield measurements were used to prove the grafting of ASA on the LDPE surface. The graft yield of modified LDPE was calculated by gravimetric measurements. The graft yield (GY) was calculated by Equation (1):

(1) $$GY[\%]=\left(\left(W_{2}-W_{1}\right)/W_{1}\right)\cdot 100\%$$

where *W*_1_ and *W*_2_ represent the weights of the LDPE samples before and after the modification.

### 2.7. Film Thickness Investigation

Thickness measurements were carried out by an F20-UVX film thickness analyzer (Filmetrics, San Diego, CA, USA) to analyze the thickness of plasma-affected and ASA-modified layers of the LDPE surface. The film thickness value was evaluated based on the differences in reflectance (%) between reference and measured samples in wavelength range of 190–1700 nm. LDPE substrate (4.5 mm thick, with refractive index of 1.5) and LDPE substrate (4.5 mm thick, with refractive index of 1.4, considering polar functional groups) were used as reference samples for the thickness measurements of plasma-treated and ASA-modified LDPE layers. Analysis of plasma-treated and ASA-modified LDPE samples was performed in air atmosphere. The spectrum was analyzed by varying the measured parameters to obtain the best fit between the theoretical and measured data using FILMeasure software, v7.19.0. Readings from five different areas were captured for each sample, and a mean value was evaluated.

### 2.8. Peel Test

A 90° peel test was performed to measure the adhesion characteristics of LDPE samples in terms of the peel resistance using a Lloyd 1K Lf plus-UTM standard testing machine (Lloyd Instruments, West Sussex, UK). Samples 19 mm in width and 6 cm in length were attached on a polypropylene tape containing poly(2-ethylhexyl acrylate) adhesive (Scotch tape). The test was undertaken with Scotch tape pressed on top of the treated LDPE surface. The unbonded end of the testing tape was peeled off at 90° at a crosshead speed of 10 mm/min. The test was stopped after 6 min when the tape was complexly detached from the LDPE surface, and 6 separate readings were carried out to obtain average values of the peeling force.

### 2.9. Surface Chemistry Characterization

Fourier transform infrared spectroscopy with attenuated total reflectance (FTIR-ATR) was used to qualitatively investigate the chemical composition changes of plasma-treated LDPE surfaces. An FTIR Spectrometer Frontier (PerkinElmer, Waltham, MA, USA) equipped with a ZnSe crystal was used for these analyses to capture data from a penetration depth of 1.66 µm. Spectra in the wavenumber range of 4000–550 cm^−1^ were obtained using an average of 8 scans, with a resolution of 4 cm^−1^.

The chemical composition changes caused by corona treatment of the LDPE surface were quantified by X-ray photoelectron spectroscopy (XPS). An AXIS XPS system (Kratos Analytical, Manchester, UK) was used for this study. The XPS system contains a spherical mirror analyzer and a delay-line detector for fast screening of the chemical composition, ensuring high spectral resolution and sensitivity. This system allows the analysis of data at a sampling depth of 1–10 nm.

### 2.10. Surface Morphology Analysis

The surface morphology of LDPE samples was analyzed by scanning electron microscopy (SEM). This technique allowed us to obtain information about surface morphology changes after each modification step. For this purpose, a Nova Nano SEM 450 microscope (FEI, Hillsboro, OR, USA) was employed. A thin Au layer a few nanometers thick was sputter-coated on the LDPE samples to obtain high-resolution images with high magnification (20,000×) and to avoid the accumulation of electrons on the measured layer.

Detailed information about the three-dimensional changes in the surface topography of the LDPE samples was obtained using atomic force microscopy (AFM). An MFP-3D AFM device (Asylum Research, Abingdon, Oxford, UK) was employed in these experiments. Scanning was carried out under ambient conditions by a silicon probe (Al reflex-coated Veeco model, OLTESPA, Olympus, Tokyo, Japan) in tapping mode in air (AC mode), allowing images with a surface area of 1 × 1 µm^2^ to be obtained. Moreover, the roughness parameter value (Ra) was calculated from AFM images obtained from Z-sensor.

### 2.11. Antibacterial Tests

A modified ISO 22196, an internationally recognized test method was used to evaluate the antibacterial activity of modified plastic materials (and other nonporous surfaces of products) to inhibit the growth of, or kill, test microorganisms [49]. The LDPE samples were first disinfected by UV radiation and then placed in sterile Petri dishes. This was followed by inoculation of the samples (25 × 25 mm^2^) using 0.1 ml of standardized bacteria suspension of *S. aureus* (CCM 4516, 1.8 × 10^6^ cfu/ml) and *E. coli* (CCM 4517, 1.4 × 10^7^ cfu/ml). The samples were covered by disinfected polypropylene foil (20 × 20 mm^2^) with 70% ethanol. Incubation of the inoculated samples was performed at 95% of relative humidity at 35 °C for 24 hours. The polypropylene foil was then removed, and LDPE samples were imprinted on plate count agar (3 times on different areas) and incubated at 35 °C for 24 hours. Then, the results were read, and the increase in the number of bacterial colonies was evaluated based on scaling from 0 to 5, where 0 represents the best antimicrobial effect, with no growth of bacteria colonies. An additional incubation at 35 °C for 24 hours was followed by final reading and evaluation of the results. All of these analyses were performed using 3 different LDPE samples to ensure reliable antimicrobial efficiency results.

## 3. Results

### 3.1. Hydroperoxide Concentration

**Figure 2:**
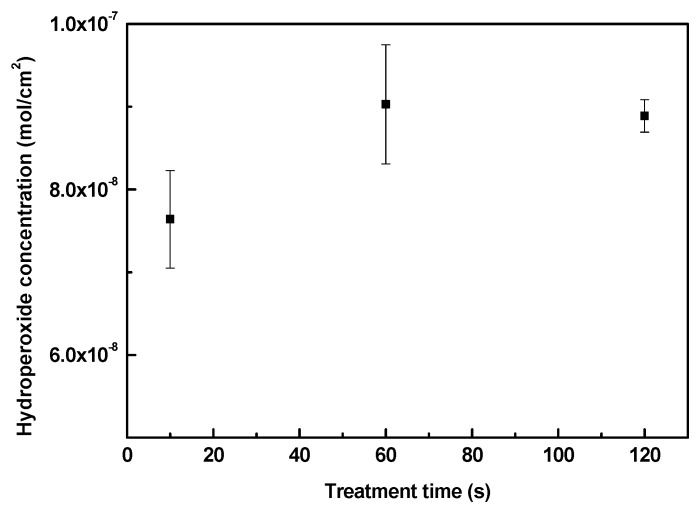
The peroxide concentration of plasma-treated LDPE samples.

Plasma treatment was used as an effective tool for generation of active species in the LDPE surface necessary for the subsequent grafting process by ASA. As the plasma treatment introduces polar functional groups onto the surface by radicalization, different kinds of functional groups can be found (mainly oxygen-containing groups). Through exposure to air during and after plasma, most of the free radicals are converted into peroxides [50]. However, it is difficult to distinguish the amount of peroxide functionalities in either the infrared (IR) spectroscopy or XPS O1s shift spectra; thus, a classic quantification method by iodometric titration according to Wagner and Thelen [51,52] was used to obtain valid concentration values. In Figure 2, LDPE samples were treated with air plasma at different exposure times. By applying iodometric titration, it was found that the hydroperoxide concentration increased as the treatment time increased from 10 s (7.6 × 10^−8^ mol/cm^2^) to 60 s (9.0 × 10^−8^ mol/cm^2^) of exposure to air plasma. An additional increase in treatment time did not lead to another increase in hydroperoxide concentration. This proves that exposing the polymer samples for a longer time does not increase the formation of hydroperoxides; thus, at the optimum time, the surface would be saturated with a certain amount of peroxides [50]. From this observation, an optimum time for achieving the maximum hydroperoxide concentration was evaluated, and LDPE samples were treated with plasma at 60 s prior to the ASA grafting process.

### 3.2. Surface Wettability Analysis

**Figure 3:**
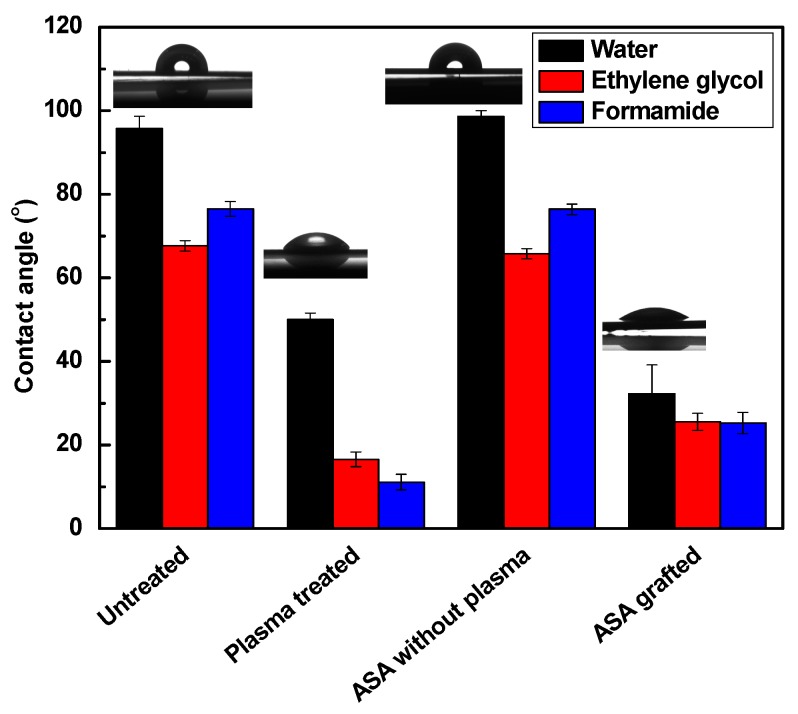
The contact angles of testing liquids on LDPE samples.

**Figure 4:**
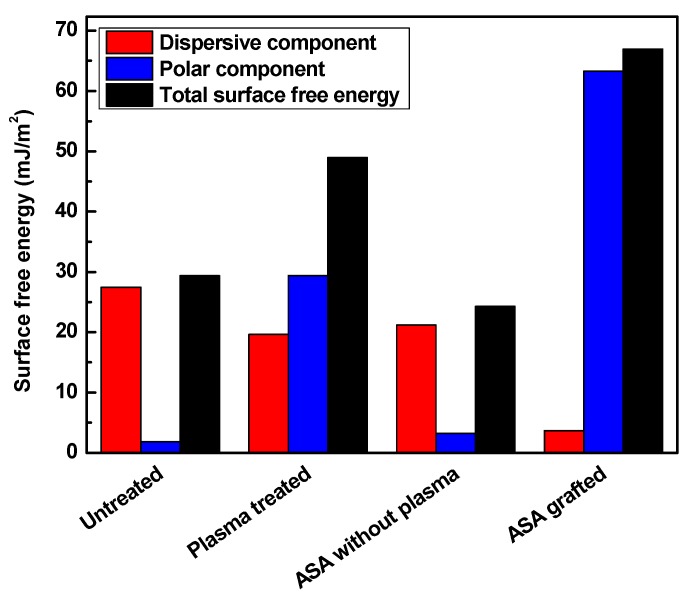
The surface free energy of LDPE samples.

The changes in the surface wettability of the modified samples were analyzed through contact angle measurements, which are shown in Figure 3 and Figure 4 and summarized in Table 1 and Table 2. Surface free energy and wettability are indicators of the ability of the liquid surface to be attached to the solid surface. This indicates that the lower the contact angle of a sample is, the higher its wettability is. To evaluate surface free energy and its components, water (surface free tension = 72.1 mN/m, polar component = 52.2 mN/m and dispersive component = 19.9 mN/m) [53], ethylene glycol (surface free tension = 48.0 mN/m, polar component = 19.0 mN/m, and dispersive component = 29.0 mN/m) [54] and formamide (surface free tension = 56.9 mN/m, polar component = 33.4 mN/m, and dispersive component = 23.5 mN/m) [53] were used. Untreated LDPE has hydrophobic properties and is chemically inert; thus, its wettability is low under basic conditions. Its water contact angel was 95.7°, with a low total surface free energy (29.3 mJ/m^2^) and insignificant polar component (1.9 mJ/m^2^). These results refer to the hydrocarbon skeleton –C–H, which has poor reactivity, and thus, no polarity was observed.

The initiation of reactions on the surface by RF plasma improved the polarity by inducing the surface through radicalization. The introduction of new oxygen-containing functional groups helped to increase the total surface free energy to 49.0 mJ/m^2^, and the contact angle of water decreased to almost half, with a value of 50.0°. The wettability increased as a result of the new polar functional groups on the surface. Plasma treatment affected the LDPE surface only at very small depth (28.2 nm), which was confirmed by film thickness measurements. Grafting of the antibacterial agent enhanced the polarity even further because of its effective side and defined structure attachment. The ASA-grafted LDPE surface using plasma treatment exhibited the lowest value for the contact angle of water (32.3°); therefore, the highest value for the total surface free energy (67.0 m J/m^2^) and its polar component (63 mJ/m^2^) was achieved. The introduced functional groups on the LDPE surface were able to effectively react and form new bonds with ASA, and the graft yield was 0.4%, indicating a multilayer formation of ASA. This was confirmed also by film thickness measurements, where the film thickness was 10.1 nm. The graft yield and film thickness analyses confirmed a formation of ASA multilayered structures on the LDPE surface. The effect of plasma treatment on the grafting of ASA onto the LDPE surface was shown, in comparison with untreated LDPE subjected to modification by ASA with subsequent thorough washing. In this case, the contact angle and the total surface free energy were similar to those of untreated LDPE.

### 3.3. Adhesion Analysis

**Figure 5:**
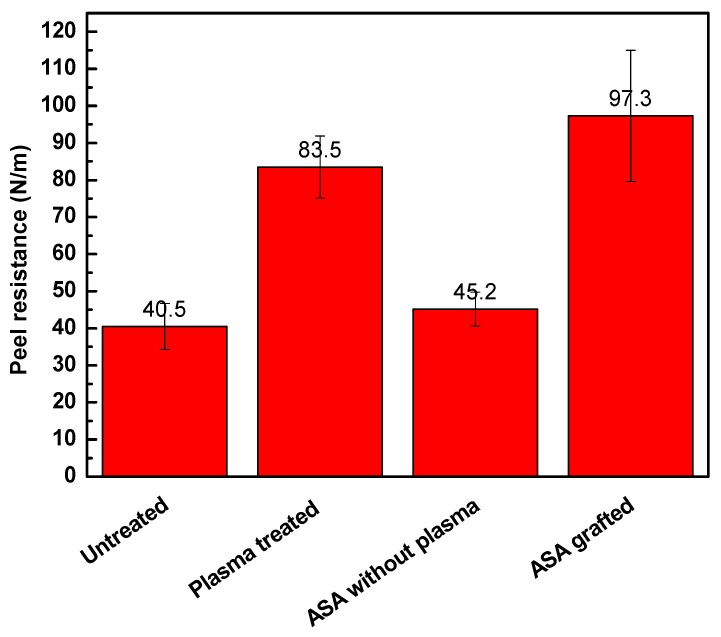
Peel resistance of LDPE samples.

Adhesion properties depend on the wettability and surface morphology (roughness) of a material surface. Roughness occurs as a result of physicochemical interactivity or chemical composition on the surface. Adhesion properties can be effectively analyzed by measurements of the peeling resistance. Figure 5 shows the changes in peeling resistance and the changes in adhesion of LDPE after plasma treatment and ASA grafting. Higher resistance induces higher adhesion, which is an outcome of a rougher surface and better wettability. The untreated LDPE surface exhibited relatively poor adhesion, and therefore, the peel resistance reached a value of 40.5 N/m because of the smooth surface and low wettability. The plasma-treated samples showed significant enhancement in the adhesion of the LDPE surface. The peel resistance increased significantly to 83.5 N/m after plasma treatment. This increase in peel resistance was affected mainly by the increase in the wettability and surface roughness caused by the incorporation of polar functional groups and etching reactions, respectively. ASA grafting onto the LDPE surface led to even higher peel resistance (97.3 N/m) because the highest wettability was achieved. ASA was also subjected to LDPE modification with and without application of plasma treatment to study the effect of plasma treatment on the covalent grafting of ASA on the LDPE surface. The untreated LDPE with ASA showed similar peel resistance (45.2 N/m) to the untreated LDPE sample, indicating the lack of ASA after thorough washing.

### 3.4. Chemical Composition Investigation

**Figure 6:**
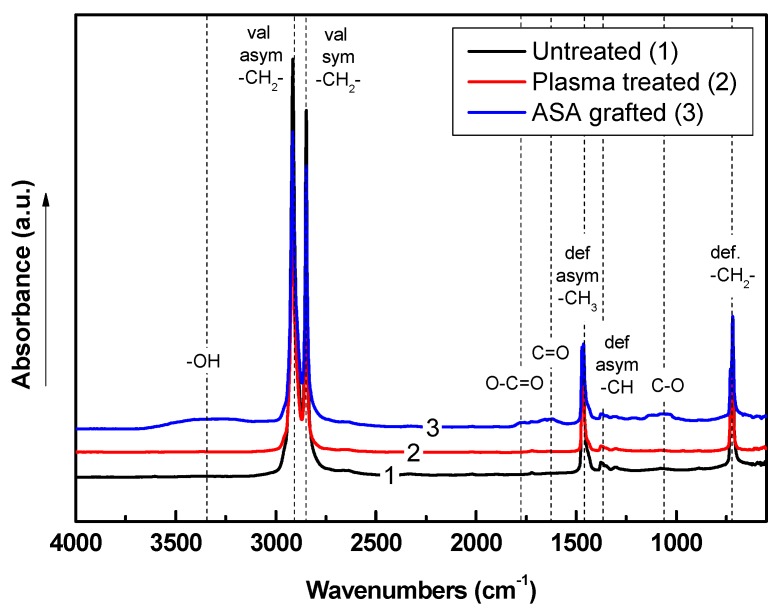
Fourier transform infrared spectroscopy (FTIR) spectra of LDPE samples.

The chemical composition of the LDPE samples after each modification step was analyzed using Fourier transform infrared (FTIR) spectroscopy. The FTIR spectra of untreated, plasma-treated, and modified LDPE samples are shown in Figure 6. The FTIR spectrum of untreated LDPE is characterized by specific absorbance bands attributed only to nonpolar hydrocarbons in the main chain and branches. These bands represent carbon–carbon and carbon–hydrogen vibrations. Thus, –C–H stretching vibrational bands are observed at 2915 cm^−1^ and 2847 cm^−1^, and bending and scissoring vibrations are observed at 1473 cm^−1^ and 717 cm^−1^, respectively. Plasma treatment of LDPE was responsible for the introduction of new functional groups by radicalizing some carbons on the polyethylene surface. This process allows the interaction between the plasma-activated surface and air elements. Oxygen-containing groups are the main functional groups following these interactions. However, these new functional groups were not clearly detected by FTIR spectroscopy because of the relatively high penetration depth of the IR beam when using a ZnSe crystal (1.66 µm) compared with the thickness of the plasma-affected layer, which was only a few tens of nm. The incorporation of new oxygen-containing groups was clearly confirmed by XPS analyses. ASA is an organic compound with ether, carbonyl, and 4-hydroxyl groups apparent in the oxygen-containing spectral region. New vibrational absorbance bands appeared in the FTIR spectrum of LDPE grafted by ASA, where –OH was represented by a broad absorbance band between 3650 cm^−1^ and 3150 cm^−1^, C=O was observed at 1623 cm^−1^, –COOH was observed at 1776 cm^−1^, and C–O–C was observed at 1060 cm^−1^. This could indicate the presence of ASA on the LDPE surface after the modification process.

**Figure 7:**
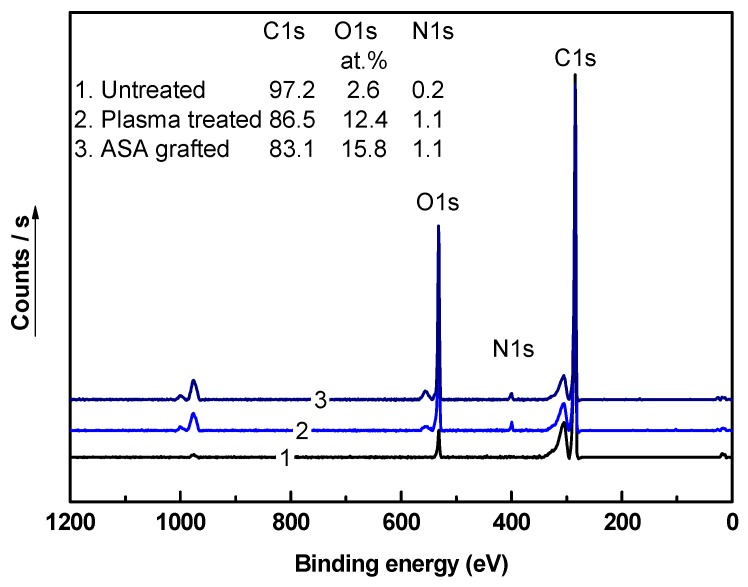
X-ray photoelectron spectroscopy (XPS) spectra of LDPE samples.

For the quantification of the chemical composition of LDPE samples, the XPS technique was employed. The XPS spectra of LDPE samples after each modification step are shown in Figure 7. The XPS spectrum of untreated LDPE consists mainly of the C1s peak, with 98 at.% at a binding energy of ~285 eV. It also contains low-intensity O1s (2.6 at.%) and N1s (0.2 at.%) peaks associated with oxygen- and nitrogen-containing functional groups, originating from processing additives or intermediates coming from air interactions with the LDPE surface. Moreover, other peaks were observed in XPS spectra, which are associated with auger electrons, and therefore they were disregarded from total atomic %. Plasma treatment of the LDPE surface was responsible for the incorporation of new functional groups, as indicated by a significant increase in the intensity of the O1s peak at a binding energy of ~530 eV, achieving a value of 12.4 at.%. In addition, the intensity of the N1s peak increased in the XPS spectrum of LDPE after plasma treatment because of the incorporation of some nitrogen-containing groups (C–N or C–NH_3_^+^). This led to a reduction in the intensity of the C1s peak to 86.5 at.% due to the removal of some carbons during etching, radicalization, and replacement with oxygen-containing groups. ASA grafting on the LDPE surface led to an increase in the at.% of the O1s peak, which was attributed mainly to the oxygen-containing groups in ASA. The ASA-grafted LDPE samples showed an O1s peak with 15.8 at.%, whereas the intensity of the C1s peak decreased to 83.1 at.%. Furthermore, the N1s intensity remained unchanged in comparison with that of the plasma-treated LDPE sample (1.1%), as ASA does not contain any nitrogen functional groups.

### 3.5. Surface Morphology Analysis

**Figure 8:**
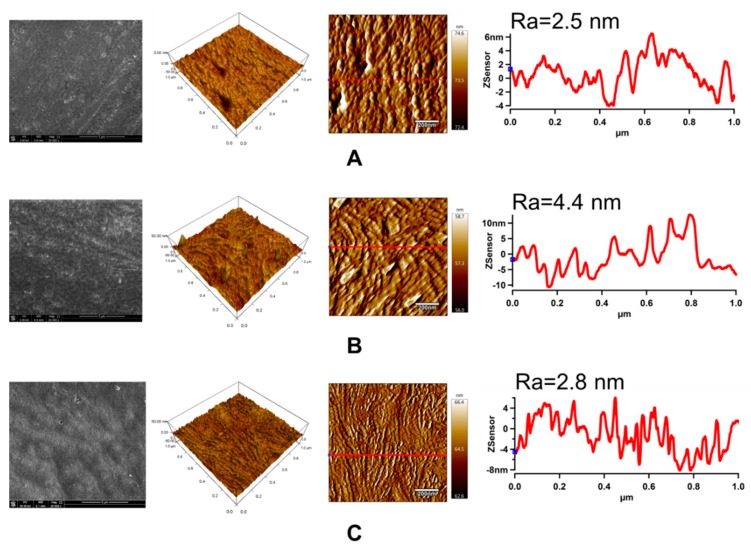
Scanning electron microscopy (SEM), 3D height. and amplitude atomic force microscopy (AFM) images with line profiles (Z-sensor) of LDPE: (**A**) untreated; (**B**) plasma-treated; (**C**) ASA-grafted. Note: Ra represents the roughness parameter.

The changes in the surface morphology and topography of the LDPE samples after each modification step were studied through SEM and AFM, respectively. SEM and AFM images of LDPE samples are shown in Figure 8. The untreated LDPE surface exhibits characteristic texture and morphology originating from the production process. Plasma treatment did not cause any significant changes in the surface morphology obtained by SEM from larger surface areas. On the other hand, the LDPE samples grafted with ASA experienced clear changes in their surface morphology, showing bulges and valleys in the functionalized regions on the surface as grafting took place.

AFM was used to determine the topographical and roughness changes (Ra) in the LDPE surface that occurred after plasma treatment and ASA grafting in the small surface area (1 × 1 µm^2^). Moreover, this technique was used to obtain line profiles, clearly indicating specific nanopattern dimensions. As reported in Figure 8, the Ra value of the untreated LDPE surface was only 2.5 nm. An application of plasma treatment led to an increase in surface roughness, while Ra increased by almost 80% to 4.4 nm. The increase in roughness can be attributed to the etching process during plasma treatment, which led to nanosurface topography changes. Grafting of ASA onto the LDPE surface resulted in a less rough surface (Ra = 2.8 nm) in the small surface area, but with a specific texture belonging to the created ASA layer.

### 3.6. Antibacterial Analysis

**Figure 9:**
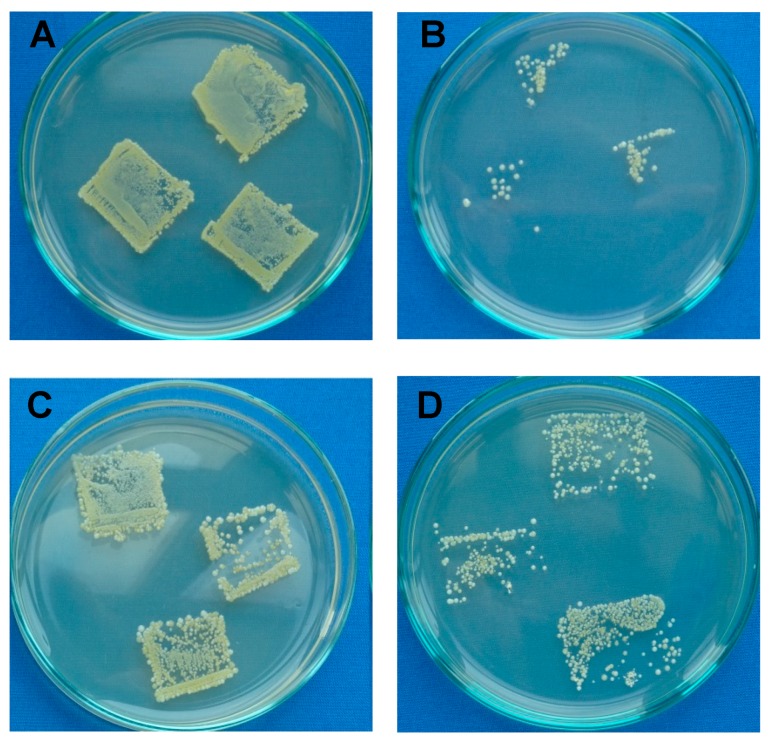
Example of total microbial counts of LDPE samples on plate count agar with inoculated bacteria: (**A**) untreated (*S. aureus*); (**B**) ASA-grafted (*S. aureus*); (**C**) untreated (*E. coli*); (**D**) ASA-grafted (*E. coli*).

The antimicrobial activities of the LDPE tested against gram-positive *S. aureus* and gram-negative *E. coli* using intensive microbial activity assays are summarized in Table 3 and are shown in Figure 9. The untreated LDPE showed no resistance or inhibition to bacterial growth. This was because of its poor inhibition properties resulting from the chemical composition of LDPE. Plasma treatment of LDPE was responsible for the low resistance to bacterial growth on the surface. On the other hand, ASA grafted on the LDPE surface showed a high ability for inhibition of *S. aureus*. ASA proved to be highly active against *S. aureus*, similar to its use alone in free form [19,23,38]. ASA could have affected the protein on the bacterial wall, affecting bacterial growth because of its ability to lower the pH and cause instability of bacterial cell membranes [55]. After ASA was grafted onto the LDPE surface, the inhibition activity against *S. aureus* was successful, with over 80%–90% total inhibition. Interestingly, the inhibition of *E. coli* growth was not as intense, although a clear reduction in colony growth and reproduction was observed, as shown in Figure 9.

## 4. Conclusions

In this study, ascorbic acid (ASA or vitamin C) was grafted onto an LDPE surface via plasma treatment in order to improve the antimicrobial effect. Plasma treatment was effectively used as a radical initiator with subsequent incorporation of ASA, which served as an antimicrobial agent, on the LDPE surface. This modification was confirmed by enhanced wettability and adhesion properties. The presence of ASA on the LDPE surface after the grafting process was confirmed by chemical composition analyses. Chemical composition and surface morphology or topography analyses were used to confirm the presence of ASA on the LDPE surface. The significant antimicrobial effect of such modified LDPE against gram-positive *S. aureus* was demonstrated, with an inhibition efficiency of over 80–90%.

## Figures and Tables

**Table 1:** The contact angles and graft yields of LDPE samples.

LDPE	Water (°)	Ethylene Glycol (°)	Formamide (°)	GY (%)	Film Thickness (nm)
Untreated (A)	95.7 (±3.0)	67.7 (±1.2)	76.5 (±1.8)	-	-
Plasma-treated (B)	50.0 (±1.6)	16.6 (±1.7)	11.1 (±1.8)	-	28.2 (±4.0)
A + ASA	98.6 (±1.4)	65.8 (±1.2)	76.4 (±1.2)	0.0	-
B + ASA	32.3 (±6.9)	25.5 (±2.0)	25.3 (±2.5)	0.4	10.1 (1.0)

**Table 2:** The surface free energy of LDPE samples.

LDPE	Dispersive (mJ/m^2^)	Polar (mJ/m^2^)	Total Surface Free Energy (mJ/m^2^)
Untreated (A)	27.5	1.9	29.3
Plasma-treated (B)	19.6	29.4	49.0
A + ASA	21.2	3.2	24.3
B + ASA	3.7	63.3	67.0

**Table 3:** Antimicrobial activity of LDPE samples.

	Increase in Bacterial Colonies ^1^	
LDPE	*S. aureus*	*E. coli*
Untreated (A)	4, 4–5, 4–5	4, 4, 4–5
Plasma-treated (B)	5, 5, 5	5, 5, 5
B + ASA	0, 1, 1	4, 4, 4

^1^ The scale for assessing the growth of bacterial colonies: 0—without growth; 1—detectable amount (single colony); 2—detectable amount (combined colony); 3—second imprint, distinguishable colonies, third imprint can be detected; 4—third imprint, distinguishable colonies; 5—overgrown, continuous growth.
